# Melanoma toolkit for early detection for primary care clinicians: a 1-year follow-up on outcomes

**DOI:** 10.3389/fmed.2024.1500216

**Published:** 2024-12-18

**Authors:** Kyra Diehl, Elizabeth Stoos, Alyssa Becker, Victoria E. Orfaly, Jacob Nelson, Jordan Gillespie, Justin Ng, Tayler Tobey, Emile Latour, Joanna Ludzik, Elizabeth G. Berry, Alan C. Geller, Heidi Jacobe, Justin Leitenberger, Danielle McClanahan, Jessica Tran, Smriti Prasad, Stephanie Mengden-Koon, Kelly C. Nelson, Ryan Petering, Alex Verdieck, Stephanie Savory, Emily H. Smith, Susan Tofte, Martin A. Weinstock, Kevin White, Oliver Wisco, Clara Curiel-Lewandrowski, Susan M. Swetter, Alexander M. Witkowski, Laura Ferris, Samantha Black, Rebecca Xu, Shuai Xu, Sancy Leachman

**Affiliations:** ^1^Department of Dermatology, Oregon Health & Science University, Portland, OR, United States; ^2^School of Medicine, Oregon Health & Science University, Portland, OR, United States; ^3^Biostatistics Shared Resource, Knight Cancer Institute, Oregon Health & Science University, Portland, OR, United States; ^4^Department of Bioinformatics and Telemedicine, Jagiellonian University Medical College, Kraków, Poland; ^5^Department of Social and Behavioral Sciences, Harvard T.H. Chan School of Public Health, Boston, MA, United States; ^6^Department of Dermatology, University of Texas Southwestern Medical Center, Dallas, TX, United States; ^7^Department of Dermatology, The University of Texas MD Anderson Cancer Center, Houston, TX, United States; ^8^Department of Family Medicine, Oregon Health & Science University, Portland, OR, United States; ^9^Department of Dermatology, University of Missouri, Columbia, MO, United States; ^10^Department of Dermatology, The Warren Alpert Medical School of Brown University, Providence, RI, United States; ^11^Providence Veteran Affairs Health Care System, Providence, RI, United States; ^12^Dermatology Health Specialists, Bend, OR, United States; ^13^Department of Dermatology, University of Arizona College of Medicine, Tucson, AZ, United States; ^14^Department of Dermatology, Stanford University Medical Center and Cancer Institute, Palo Alto, CA, United States; ^15^Department of Dermatology, University of Pittsburgh, Pittsburgh, PA, United States; ^16^Brigham and Women's Hospital, Harvard Medical School, Boston, MA, United States; ^17^School of Medicine, Northwestern University, Chicago, IL, United States; ^18^Knight Cancer Institute, Oregon Health & Science University, Portland, OR, United States

**Keywords:** education, melanoma, primary care, skin cancer, skin neoplasms, training

## Abstract

**Introduction:**

Primary care providers or clinicians (PCPs) have the potential to assist dermatologists in screening patients at risk for skin cancer, but require training to appropriately identify higher-risk patients, perform skin checks, recognize and biopsy concerning lesions, interpret pathology results, document the exam, and bill for the service. Very few validated dermatology training programs exist for PCPs and those that are available focus primarily on one emphasis area, which results in variable efficacy and single-topic limited scope.

**Methods:**

We have created a free, online, continuing education program (Melanoma Toolkit for Early Detection, MTED) that allows learners to choose from a variety of multimedia tools (image recognition, videos, written material, in-person seminars, self-tests, etc.) that suits their learning style and time availability. Here we present the toolkit, the development and validation of the curriculum, and report on 1-year outcomes of a nested survey study. Because the goal of the program is to maximize participation by allowing PCPs to tailor their experience to their own needs and interests, the majority of participants did not complete every element of the program.

**Results:**

A total of 8,683 PCPs have accessed at least one element of the toolkit from 2019–2024. Participants completed a pre-survey, online training module, and post-survey that included self-reported screening behaviors, changes in confidence, and malignant and benign lesion categorization based on clinical images. A total of 139 pre-surveys and 92 post-surveys were completed, including 55 matched participants that completed both the pre- and post-training surveys. There were significant improvements in PCP confidence (*P* < 0.001) and malignant (*P* < 0.001) and benign image (*P* = 0.029) identification respectively.

**Discussion:**

PCPs may serve as a valuable aid in skin cancer screening efforts, but additional studies are needed to evaluate the impact of these curricula in clinical practice.

## Introduction

There are not enough dermatologists to permit screening of all of the patients who have an increased risk of skin cancer (1, 2). One possible solution to this shortage is for PCPs to incorporate skin examinations into their practice when appropriate, as they already play a key role in assessing patients' self-identified lesions of concern. Additionally, important skin cancer education, such as the “ABCDEs” of melanoma (3, 4), can be easily integrated into their patient visits. However, PCPs should not be screening everyone. The United States Preventive Services Task Force (USPSTF) is a national, evidence-based advisory group that makes recommendations regarding the appropriateness of using screening examinations/tests by PCPs. The USPSTF does not recommend skin cancer screening of the general public by PCPs but does recommend that high-risk patients be screened (5). Unfortunately, the USPSTF does not provide a clear definition of “high-risk,” though efforts have been made in the dermatology community to do so (6). In addition to a lack of guidance with respect to which populations to screen, many PCPs have not had much training in skin examination (or have had it many years ago), and do not have confidence that they can identify melanoma successfully or have the time to perform skin examinations in their busy clinics (7–11).

One way to address these issues is to provide free, online education to PCPs who want to incorporate skin cancer screening into their practice, but need more education, expertise, or time-saving strategies. Australia has a well-established culture of PCP-based melanoma screening, with PCPs managing the majority of all melanomas in the country (12–14). This suggests that an educational toolkit that addresses the issues above might recruit more PCPs in the USA and improve access to screening. Further, educational models that emphasize behavioral change produce more desirable outcomes compared to those focused only on improving knowledge (15). In an effort to produce a lasting practice change, we developed a toolkit of educational materials aimed at improving participant knowledge, confidence, and behaviors.

## Methods

### Needs assessments

The Oregon Health & Science University (OHSU) institutional review board approved the educational toolkit curricula and the nested survey study (STUDY00019372). A needs assessment performed by the Oregon Echo Network (OEN) found that dermatologic care is one of the most desired educational opportunities by PCPs and that PCPs consider dermatologists to be one of the most needed subspecialists in the state. In addition to knowing that dermatology is an important need, we wanted to know what obstacles were preventing PCPs from performing skin examinations. To find this information, we performed an additional, community-based needs assessment of Oregonian PCPs through informal focus-group sessions at seven hospitals and clinics statewide. These sessions involved ~60 PCPs and took place during Continuing Medical Education (CME) events hosted by the War On Melanoma™ (WoM) community outreach program. We also distributed online surveys, receiving responses from 218 PCPs in Oregon and Texas. Additionally, we conducted a literature review that included findings from a national survey. Results of our needs assessment confirmed that some PCPs are overbooked and over-tasked, but also demonstrated that some PCPs have the capacity and desire to do skin examinations. Clinicians had a high level of concern about missing melanoma in their patient population and indicated an interest in learning how to accurately detect melanomas. They expressed diverse preferences in the way they wanted to receive the education, including in-person venues, traditional text-based material, games, image-based self-tests, and videos.

### Toolkit conceptual goals

The overarching goal of the toolkit was to improve skin cancer detection by increasing PCP confidence, screening attitudes, and clinical knowledge and diagnostic skills. The format identified by our needs assessment as most convenient for learning are online, asynchronous learning modules that incorporate video demonstrations and images, allowing participants to review and practice with immediate feedback. Evidence-based learning strategies were employed, including case-based discussions, learner-paced instruction, practice with immediate feedback, and segmented topics with a clear link to learning objectives. The needs assessment clearly indicated that the toolkit also needed to be flexible and tailored to the needs of individual PCPs.

### Learning management system selection

Learning Management Systems (LMS) are software applications that permit online delivery and tracking of educational courses, but each system has its own set of strengths and weaknesses. We initially developed our toolkit in Articulate^®^, an LMS that is very flexible and permits testing and tracking of users. A downside of Articulate^®^ was that it required external OHSU learners to pay for usage, so we decided to trial OHSU's LMS, Compass. However, Compass requires users to create an account and log-in each time they access the training. During our piloting period, we received feedback that logging into an LMS was an obstacle, and learners preferred the training be located on an accessible web-page. For these reasons, we moved the toolkit to Articulate Review^®^, which is less expensive than Articulate^®^ and made the toolkit easily accessible online. Importantly, a limitation of Articulate Review^®^ is that it does not allow for data tracking and analysis. Therefore, we were unable to track learner progress, including which parts of the toolkit were accessed by each participant. The final toolkit was piloted with all three of these LMS systems [see Orfaly et al. (16)], and the survey data presented here includes data from the final LMS, Articulate Review^®^, following the completion of the pilot study.

### Toolkit testing and revision

Once the first version of the toolkit was designed, the pilot study was performed and leaders in dermatology and primary care at OHSU, Stanford, University of Pittsburgh Medical Center, and University of Texas (UT) Southwestern were asked to assess the quality and useability of the toolkit (16). Based on these assessments, a second iteration of the toolkit and associated surveys was created. A summary of design changes to the final toolkit included:

1. Adjusted length and content: based on feedback that it was too long and contained information not relevant to PCPs—the links to the additional INternet curriculum FOR Melanoma Early Detection (INFORMED) modules were removed. These are planned to be added back as a second, more in-depth additional learning module in the next iteration, making a “Level 1” and “Level 2” to meet the needs and interests of learners.
2. Added electronic medical record (EMR) tools: dermatology and primary care stakeholders recommended including EMR tools to facilitate and optimize implementation of toolkit materials into clinical practice. We developed and added a resource sheet on risk factors, as well as a SmartPhrase for Epic which was linked for download by OHSU users and included as a script for external users to add to their own EMR. This includes yes/no questions for a scored risk assessment, stratification, and prioritization. We also created a screening guideline sheet with risk factors, common International Classification of Diseases-Tenth Revision (ICD-10) codes, and helpful tips for easy printable references.
3. Incorporated patient-directed materials for adults and children: another recommendation was to add patient education materials that could be printed or included in after-visit summaries when time constraints made it difficult to perform a skin exam at that clinic appointment. In this way, clinicians can empower patients to learn more, perform high-quality self-examinations, and identify lesions of concern that could be expedited for dermatology review. To facilitate this, we included patient education materials in the Resource section of the Toolkit and created an After-Visit Summary draft for adult and pediatric patients that can be added to EMRs.
4. Enhanced diversity and skin of color (SOC) MATERIALS: ANOTHER IMPORTANT feedback was the lack of diversity of skin color and specific recommendations for patients with SOC. To meet this need, we collaborated with Samantha Black, a Fellow at UT Southwestern, who created a video module on skin exams for SOC patients in the Skin Exams section, as well as an additional learning module on acral melanoma in the Visual Identification section. We also added questions to the pre- and post-surveys asking about attitudes toward screening SOC patients. Additionally, we found new images with more diverse skin colors and updated the Image Identification section of the pre- and post-surveys.
5. Refined image visualization and identification: as feedback, dermatologists added that the ability to determine whether a lesion is malignant is based on patient history and risk factors, in addition to visual perception. In response, we added case history notes in the case-based learning slides and included more images in the Image Identification slides that could not be identified from visualization alone. The pilot data was reviewed for the Image Identification sections of the pre- and post-tests. Images that were outliers were identified as “too easy” or “too difficult” and removed. For images that showed multiple lesions, an arrow was added to specify which lesion was being asked about. A national team of expert dermatologists met and reviewed the images to decide the best ones to include as representative of each lesion type. The pre- and post-surveys were updated with new images. This group also decided that because the critical decision-making step in early melanoma diagnosis is the decision to biopsy, the preferred outcome measure for PCPs is how closely their decision to biopsy matched expert dermatologists.

### Toolkit final design

**Figure 1:**
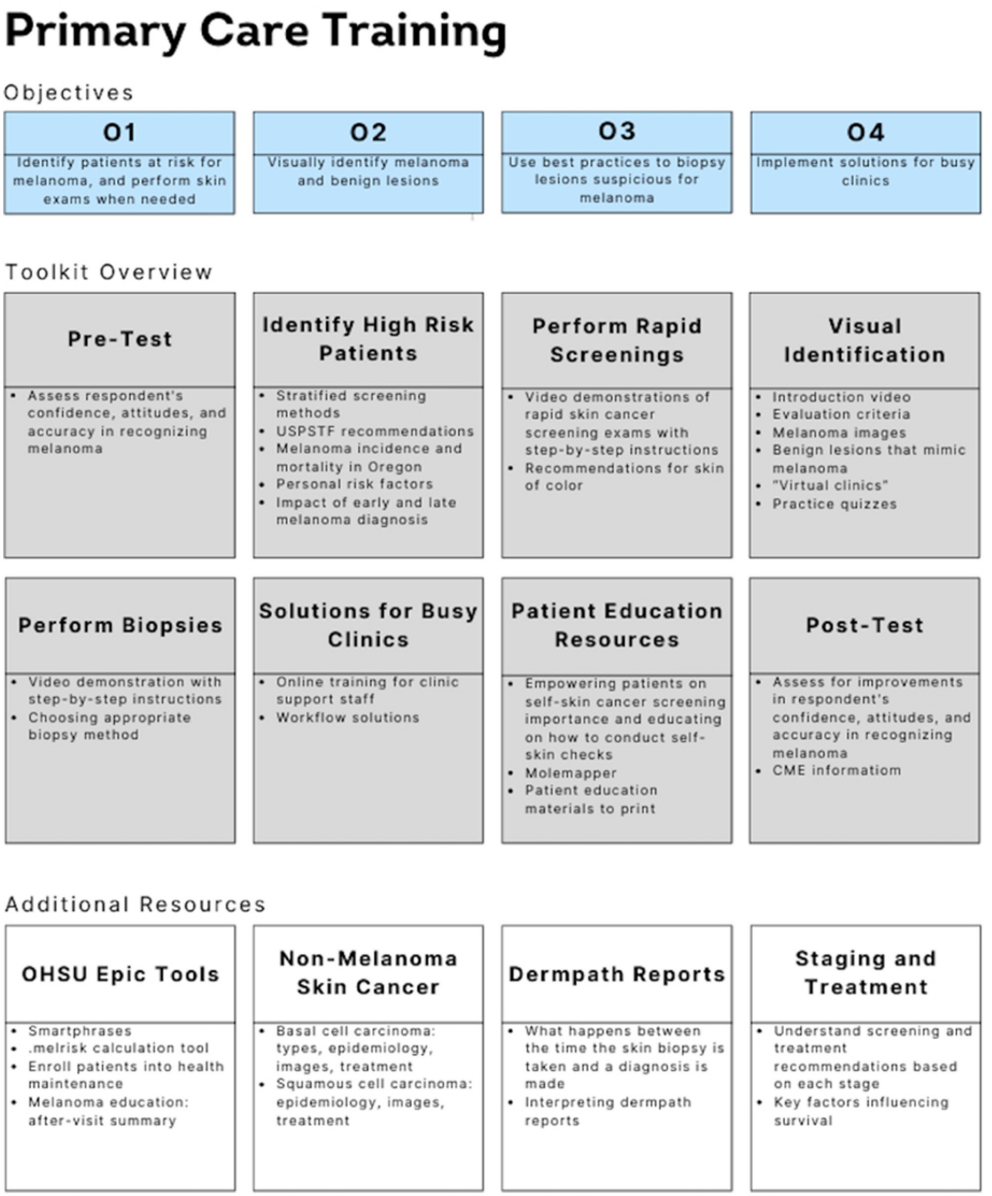
Schematic detailing the four objectives of the melanoma training, an overview of the different parts of the toolkit (including a pre-test and post-test), and additional resources provided to participants. The curriculum covers identification of high-risk patients, how to perform a skin screening, visual identification of benign and malignant lesions, how to perform skin biopsies, solutions for busy clinics, and patient education resources. The toolkit is aimed to provide information and resources to equip the user to provide screenings for skin cancer, detect melanoma early, and empower patients with provided educational resources.

The final toolkit was built and delivered using the online e-learning platform Articulate Review^®^ and can be accessed at https://www.ohsu.edu/war-on-melanoma/melanoma-early-detection-toolkit (Supplementary Appendix A). The training consists of six modules (Figure 1), which can be completed in any order to allow flexibility and freedom for each participant to gain the information they want.

### Curriculum design and content

Our toolkit incorporates multimedia online instruction with pre-and post-training tests to determine whether participation in the program changed knowledge, confidence, or intended practice behaviors. The content of the curriculum was inspired by three previously published PCP curricula, including: (1) the Skin Cancer Research to provide Evidence for Effectiveness of screening in Northern Germany (SCREEN) project, a successful state-mandated screening initiative (17, 18), (2) the INternet curriculum FOR Melanoma Early Detection (INFORMED) curriculum (19), and (3) a targeted Visual Perception Training (VPT) program (20). Our content contains elements of all three programs; however, unlike SCREEN, our training doesn't have any in-person training or clinician screenings because those elements were supported by the German National Health Service, which is unavailable in the USA. Similar to INFORMED, our program is interactive, web-based, and designed to facilitate optimal, less labor-intensive distribution, and provide flexibility for the learner. We also collaborated with the developers of the targeted VPT program to incorporate its concepts with a customized visual identification module that can be tested through quizzes with instant feedback. To further accommodate the diverse needs and preferences of learners, we opted to create a “Toolkit” rather than a traditional curriculum. This approach allows users to choose the resources that best fit their individual learning needs.

### Pre- and post-training survey design

Participants took a pre-survey before beginning any element of the curriculum and a post-survey immediately after completion. The pre-survey respondents were separated into licensed healthcare clinicians (who answered all survey questions) and students, who only completed the visual identification and confidence portions (due to their lack of experience as practicing clinicians, preventing them from being able to answer the other portions). However, every respondent could answer all post-survey questions. The pre- and post-surveys assessed participants' screening behaviors and attitudes, confidence in identifying melanoma, barriers to performing skin exams, and knowledge about SOC. Results from the survey were matched by respondents and analyzed for significance and data trends. Descriptive statistics (counts and percentages) were used to describe the demographic characteristics of the respondents. Changes in confidence from pre- to post- surveys were assessed using paired *t*-tests.

**Figure 2:**
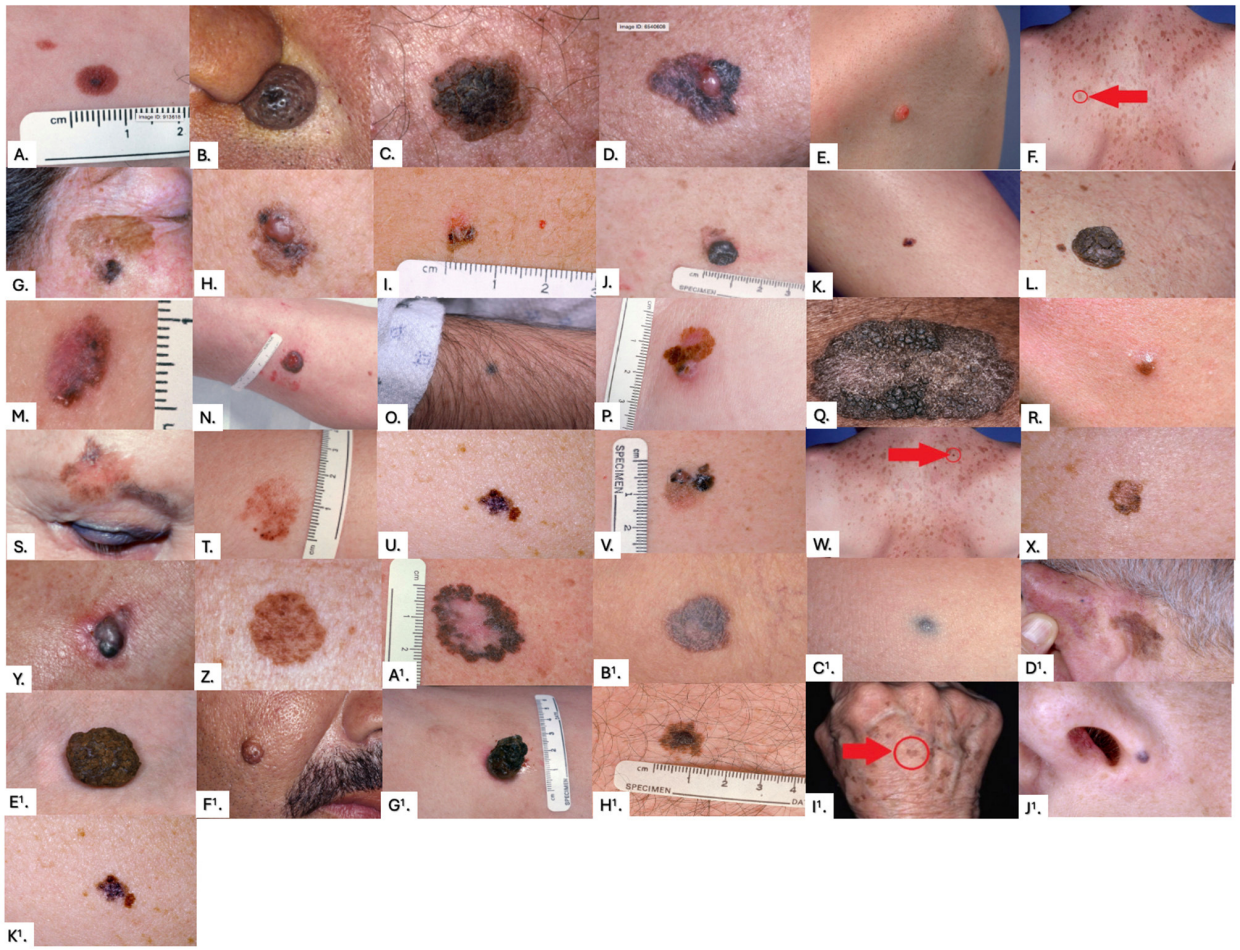
Images from the pre-training and post-training surveys. Correct answers were determined by universal agreement among the six dermatologists, with any disagreement resulting in the lesion being labeled as “indeterminate.” Correct answers were as follows: **(A)** Monitor, **(B)** Monitor, **(C)** Monitor, **(D)** Biopsy, **(E)** Indeterminate, **(F)** Monitor, **(G)** Biopsy, **(H)** Biopsy, **(I)** Indeterminate, **(J)** Biopsy, **(K)** Indeterminate, **(L)** Monitor, **(M)** Biopsy, **(N)** Biopsy, **(O)** Indeterminate, **(P)** Biopsy, **(Q)** Indeterminate, **(R)** Indeterminate, **(S)** Biopsy, **(T)** Indeterminate, **(U)** Biopsy, **(V)** Biopsy, **(W)** Monitor, **(X)** Indeterminate, **(Y)** Biopsy, **(Z)** Indeterminate, **(A1)** Biopsy, **(B1)** Indeterminate, **(C1)** Indeterminate, **(D1)** Indeterminate, **(E1)** Monitor, **(F1)** Monitor, **(G1)** Indeterminate, **(H1)** Indeterminate, **(I1)** Monitor, **(J1)** Indeterminate, **(K1)** Biopsy.

On the pre- and post-surveys, participants were asked to rate the probability (%) of 37 lesion images on their likelihood of being a melanoma (Figure 2). The images were the same on both surveys. Respondents' answers were compared to those of six pigmented lesion expert dermatologists using a two-sample *t*-test. Changes in test scores from pre- to post- surveys were assessed with paired *t*-tests. Analysis was performed using R: A Language and Environment for Statistical Computing (21). *P* < 0.05 was considered to be statistically significant.

## Results

Between 2019 and 2024, the toolkit was viewed a total of 11,244 times by 8,683 unique users. Overall, 139 participants took the pre-survey and 92 took the post-survey. Of those, 55 participants who completed both components were matched and included for analysis. Table 1 summarizes the participant demographics and pre- and post-training survey results. Of the six toolkit modules offered, the Patient Education Resources section was completed by the largest percentage of respondents (50.9%), followed by the Visual Identification portion (41.8%). There were 33 (60.0%) licensed healthcare clinician respondents, and 22 (40.0%) students. The majority of clinicians (60.6%) were in their early-careers with only 1–5 years of practice. Although PCPs acknowledged the importance of melanoma screening, the majority were not screening for or educating their patients about skin cancer, with 78.8% of clinicians reporting they “never” or only “sometimes” have their patients change into gowns to examine their skin. Moreover, after participating in our training, PCPs identified more barriers to performing skin exams, with a significant increase in the number of respondents who saw limited time as a barrier to screening (*P* < 0.001). However, our curriculum was successful in providing SOC education, demonstrating a 36.9% post-training increase in the number of respondents who felt they had received adequate education on melanoma in SOC patients.

**Table 1:** Primary care physician demographics, screening behaviors, and attitudes pre- and post-training.

**A. Self-reported completion of toolkit sections**	
**Section completed**	***n*** **(%)**
Section 1: high-risk patient identification	22 (40.0)
Section 2: how to perform rapid screenings	22 (40.0)
Section 3: visual identification of benign and malignant skin lesions	23 (41.8)
Section 4: biopsy instruction	19 (34.5)
Section 5: solutions for busy clinics	18 (32.7)
Section 6: patient education resources	28 (50.9)
Total responses	55 (100)
**B. Respondent demographics**	
**Credentials**	***n*** **(%)**
MD	12 (21.8)
DO	3 (5.5)
PA	3 (5.5)
NP	9 (16.4)
Resident	2 (3.6)
Student	22 (40.0)
Other	4 (7.3)
Total responses	55 (100)
**Years in practice**	***n*** **(%)**
1–5	20 (60.6)
6–10	2 (6.1)
11–15	2 (6.1)
16–20	3 (9.1)
21 or more	6 (18.2)
Total responses	33 (100)
**Region of practice**	***n*** **(%)**
Portland metro	9 (27.3)
Willamette valley	3 (9.1)
Eastern Oregon	1 (3.0)
Southern Oregon	8 (24.2)
North coast	4 (12.1)
South coast	5 (15.2)
Texas	3 (9.1)
Total responses	33 (100)
**C. Estimated racial distribution of respondent's patients**						
**Patient race**	**Very few** ***n*** **(%)**	<**50%** ***n*** **(%)**	>**50%** ***n*** **(%)**	**Almost all** ***n*** **(%)**	**Don't know** ***n*** **(%)**	**Total responses** ***n*** **(%)**
African American	16 (50.0)	7 (21.9)	4 (12.5)	0 (0.0)	5 (15.6)	32 (100)
Asian or Pacific Islander	18 (56.2)	8 (25.0)	1 (3.1)	0 (0.0)	5 (15.6)	32 (100)
Hispanic	2 (6.2)	20 (62.5)	5 (15.6)	0 (0.0)	5 (15.6)	32 (100)
Native American	23 (71.9)	3 (9.4)	1 (3.1)	0 (0.0)	5 (15.6)	32 (100)
White	0 (0.0)	4 (12.1)	13 (39.4)	12 (36.4)	4 (12.1)	33 (100)
**Behavior**	**Never** ***n*** **(%)**	**Sometimes** ***n*** **(%)**	**Often** ***n*** **(%)**	**Always** ***n*** **(%)**	**Total responses** ***n*** **(%)**
Examine exposed skin of patients at risk for melanoma	5 (15.2)	12 (36.4)	10 (30.3)	6 (18.2)	33 (100)
Have patients at risk for melanoma change into a gown and examine their entire skin	16 (48.5)	10 (30.3)	4 (12.1)	3 (9.1)	33 (100)
Advise use of daily sunscreen	5 (15.2)	11 (33.3)	13 (39.4)	4 (12.1)	33 (100)
Advise use of sun-protective clothing	9 (27.3)	11 (33.3)	11 (33.3)	2 (6.1)	33 (100)
Advise seeking shade or avoiding the sun	7 (21.2)	12 (36.4)	10 (30.3)	4 (12.1)	33 (100)
Show patients how to do self-skin exams	18 (54.5)	11 (33.3)	3 (9.1)	1 (3.0)	33 (100)
Explain melanoma warning signs or the ABCDE criteria for melanoma detection	8 (24.2)	18 (54.5)	5 (15/2)	2 (6.1)	33 (100)
**E. Changes in attitudes and behaviors regarding skin cancer screening and detection**		
**Barrier to performing a complete skin exam during annual wellness visits**	**Pre-training** ***n*** **(%)**	**Post-training** ***n*** **(%)**
Limited time in health maintenance visits	23 (41.8)	40 (72.7)
Performing a full skin exam is not a priority	9 (16.4)	12 (21.8)
Lack of adequate training in this area	13 (23.6)	18 (32.7)
Patient population not generally at high risk	1 (1.8)	2 (3.6)
Lack of USPSTF recommendation	9 (16.4)	6 (10.9)
Reimbursement issues	4 (7.3)	9 (16.4)
Patient embarrassment or reluctance	8 (14.5)	16 (29.1)
I don't have any barriers	3 (5.5)	10 (18.2)
**Next step after finding a skin lesion concerning for melanoma on a patient**	**Pre-training** ***n*** **(%)**	**Post-training** ***n*** **(%)**
Biopsy the lesion	9 (27.3)	16 (29.1)
Refer to a dermatologist	23 (69.7)	38 (69.1)
Other	1 (3.1)	1 (1.8)
Total responses	33 (100)	55 (100)
**Importance of melanoma screening and education in their practice (1** = **not important, 5** = **extremely important)**	**Pre-training** ***n*** **(%)**	**Post-training** ***n*** **(%)**
1	3 (5.5)	1 (1.8)
2	3 (5.5)	1 (1.8)
3	15 (27.3)	12 (21.8)
4	12 (21.8)	16 (29.1)
5	22 (40.0)	25 (45.5)
Total responses	55 (100)	55 (100)
**Beliefs of skin cancer in patients of skin of color (SOC)**		**Pre-training** ***n*** **(%)**	**Post-training** ***n*** **(%)**
Patients of SOC should be screened for melanoma	Agree	54 (98.2)	52 (96.3)
	Disagree	1 (1.8)	2 (3.7)
	Total responses	55 (100)	54 (100)
Patients of SOC hold a significant risk for development of melanoma	Agree	41 (74.5)	43 (79.6)
	Disagree	14 (25.5)	11 (20.4)
	Total responses	55 (100)	54 (100)
Patients of SOC should be educated on sun habits regularly	Agree	54 (98.2)	53 (96.4)
	Disagree	1 (1.8)	2 (3.6)
	Total responses	55 (100)	55 (100)
Educating patients of SOC on their risk for developing melanoma is worthwhile	Agree	52 (94.5)	53 (96.4)
	Disagree	3 (5.5)	2 (4.6)
	Total responses	55 (100)	55 (100)
To date, I have received adequate education on melanoma in patients with SOC	Agree	16 (29.1)	35 (66.0)
	Disagree	39 (70.9)	18 (34.0)
	Total responses	55 (100)	53 (100)
**F. Post-training attitudes toward skin cancer screening**	
**Prompt to perform a complete skin exam**	***n*** **(%)**
Patient requested a check of a suspicious lesion	50 (90.9)
Patient has a personal or family history of melanoma	46 (83.6)
Patient is fair skinned/light haired	38 (69.1)
Patient's chief complaint is skin related	36 (65.5)
I always examine the skin for melanoma in an annual physical	20 (36.4)

### Lesion identification: accuracy and confidence

**Figure 3:**
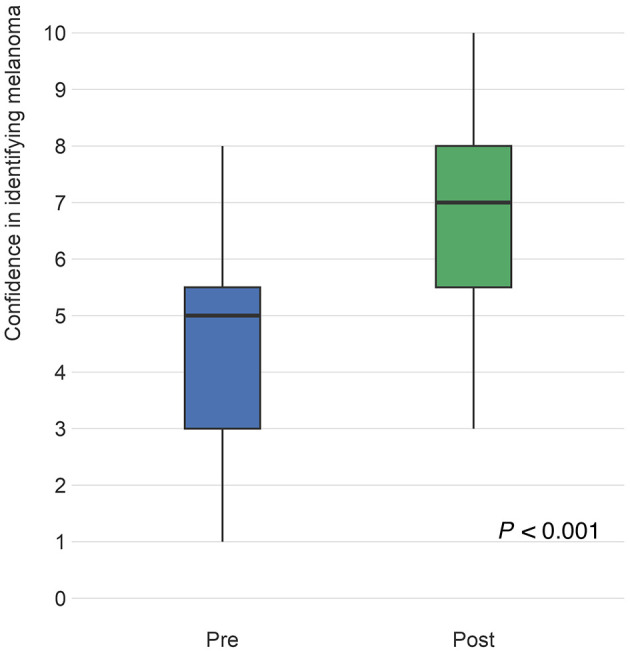
Questions intended to evaluate triage accuracy presented an image of a skin lesion and asked the respondent to rate the “probability (%) or likelihood that the image represents melanoma” on a scale of 0–100%. Participant answers were compared to the “gold standard” ratings of six pigmented lesion expert dermatologists, with malignant images scoring higher and benign images scoring lower. The figure compares the distribution of pre-test (blue) responses to post-test (green) responses for malignant and benign images. Differences from pre to post were analyzed using linear mixed models to account for within respondent variation as well as pre- to post- variation of responses. From pre- to post-training, the ratings for malignant images increased by an average of 5.3 percentage points (95% CI: 3.6–6.9), which was statistically significant (*P* < 0.001) and for benign images, likelihood a lesion was malignant significantly decreased on average by 2.9 (95% CI: 0.3–5.5) percentage points (*P* = 0.029).

PCPs and six expert dermatologists set a threshold of certainty that they required to perform a biopsy on a specific melanocytic lesion of concern and then rated a series of images based on their perceived likeliness of being melanoma. This allowed each lesion to be triaged to “biopsy” or “no biopsy” categories. PCP triage accuracy was determined relative to the “gold-standard” ratings of the expert dermatologists. Appropriate triage of malignant lesions significantly improved post-training by an average of 5.3% (95% CI: 3.6–6.9; *P* < 0.001). Similarly, appropriate triage of benign images significantly improved by an average of 2.9% (95% CI: 0.3–5.5; *P* = 0.029; Figure 3).

**Figure 4:**
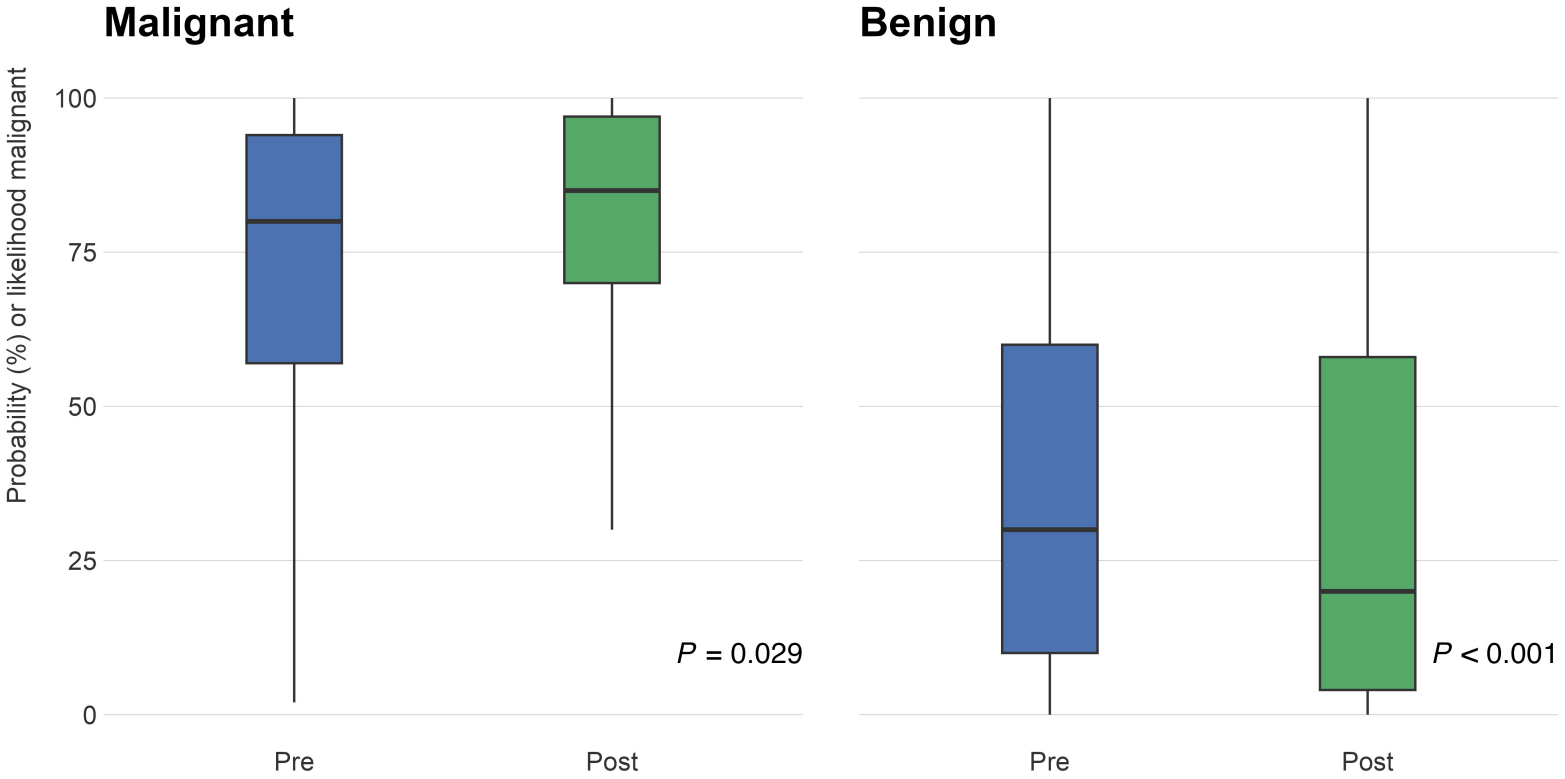
Participants (*n* = 55) were asked to rate their confidence in identifying melanomas on a scale of 1–10 (1 being “uncertain” and 10 being “certain”) on the pre- and post-surveys. The distribution of responses on the pre-test (blue) and post-test (green) are shown above. From pre- to post-training surveys, confidence ratings increased by 2.1 points (95% CI: 1.6–2.6; *P* < 0.001).

PCPs were asked to rate their confidence in identifying melanoma on a scale of 1–10, with 1 being “uncertain” and 10 being “certain.” Following participation in the curriculum, confidence levels significantly increased by 2.1 points (95% CI: 1.6–2.6; *P* < 0.001; Figure 4).

## Discussion

This project aimed to provide a well-validated and rigorously measured skin cancer education curriculum for PCPs across Oregon and provide a framework for successful PCP education initiatives to be replicated elsewhere in the future. After the pilot study, our curriculum was validated through needs assessments, careful revisions that incorporated user feedback, and expert testing. The variety of tools available in the educational toolkit permitted learners to self-select the learning methods that are best suited to their practice, schedule, and learning style. The curriculum was purposefully delivered through online multimedia tools to account for time sensitivity and unique knowledge level of each user. Providing meaningful education in a time efficient manner is crucial to engaging busy healthcare clinicians, and the flexible “toolkit” design of our web-based resources allowed participants to engage in the content most relevant to their pre-existing knowledge base. A systematic review on PCP skin cancer education programs found that the interventions that were successful in effecting behavior change used online interactive formats, while unsuccessful interventions were brief and passive (22). We similarly had success with an online formatted curriculum, with significant increases in our cohort's ability to identify malignant and benign lesions.

PCPs in our cohort demonstrated an improved ability to differentiate between malignant and benign skin lesions, signifying their potential to aid dermatologists in skin cancer detection. However, the impact this will ultimately have in improving patient access is yet to be determined in Oregon and is the subject on the ongoing War On Melanoma program. However, in Australia, a well-established culture of PCP-based melanoma screening serves as a prime example of how PCP trainings can affect the healthcare system and patient outcomes (13). An analysis of Australian skin screening clinical outcomes reported that PCPs have an 86% specificity for detecting melanoma through FBSEs, which is comparable to that of other screening tests, including mammography for breast cancer (94%−97%) (23). However, they also demonstrated a greater improvement in confidence that could signal a tendency toward misdiagnosis.

Our curriculum also aimed to improve PCP's confidence in conducting screenings, while avoiding the risk of fostering excessive confidence levels. Overconfidence without a corresponding improvement in the ability to recognize signs of malignancy could result in malignant lesions being overlooked, leading to missed or delayed melanoma diagnoses. Conversely, low levels of confidence could lead to over-referral of benign lesions for evaluation by dermatologists, which could worsen access within dermatology and potentially cause emotional distress and unnecessary scarring for the patient. Our results are promising because we have been able to improve both the accuracy and the confidence levels of PCPs, but the downstream effects of our toolkit on referral for biopsy have not yet been determined. However, it has been shown by others (24–26) that the diagnostic accuracy of PCPs can be improved without a rise in unnecessary referrals, suggesting that we may see positive impact of these educational interventions. Additional real-world implementation studies will be needed to determine the overall impact.

Given that racial and ethnic minorities are more likely to receive later-stage melanoma diagnoses (27), our curriculum emphasized the importance of screening patients from diverse backgrounds, providing targeted education on identifying skin cancer in SOC populations and focusing on high-risk areas in people with more darkly pigmented skin. PCPs in our cohort reported seeing very few patients with SOC, which suggests they may be less familiar with identifying skin lesions in these individuals, further underscoring the importance of the education we provided. Our curriculum led to an increase in the number of PCPs who felt they had received adequate education on melanoma in SOC patients. Not only does this demonstrate the success of our curriculum's SOC skin cancer education, but also indicates that our curriculum introduced knowledge that was not covered in our participants' initial medical educations.

### Limitations

The primary limitation of this study is the small sample size of participants who completed the pre- and post-surveys as well as the significant drop-off in the number of participants who either just viewed the curriculum or engaged with only parts of it, compared to those who completed the entire curriculum. This challenge is seen in similar PCP online training studies due to time constraints preventing them from being able to complete educational tasks outside of their busy practices. For example, a short (2-h length) self-paced PTSD course reported only 33% study completion by participants despite an additional incentive fee and CME credit (28). Our learner-centered curriculum was designed to cater to the user, allowing them to engage in specific modules they found helpful. Although this created a more positive experience for the learner, it likely resulted in a smaller sample size of highly motivated participants that were interested in engaging with more of the curriculum, which could produce an enrollment bias. Additionally, the large number of PCCs who accessed the toolkit but did not complete the surveys may be attributed to time constraints or a perceived lack of relevance to their practice. These factors should be explored in future studies to better understand barriers to participation and engagement, and to identify strategies for improving completion rates.

Additionally, the relatively modest improvement in triage rates may reflect a limitation in the specific images utilized in this study. We opted to include less obvious, indeterminate-appearing lesions as well as classic benign- and malignant-appearing lesions. This choice was made to try and simulate a situation that was representative of the lesions PCPs may see in the real-world; however, in return this made the test more difficult, which may have led to less marked improvement in our results. Furthermore, participants demonstrated strong baseline performance, leaving less room for improvement. This may be explained by the fact that our cohort consisted of PCCs with prior healthcare training and therefore a foundational knowledge of skin cancer. If this curriculum was administered to the general public, who typically lack such knowledge, we might observe more significant improvements in triage rates. Lastly, our test asked participants to rate their perceived likelihood of a lesion being melanoma. The wording of this question may have introduced ambiguity into their responses. To enhance clarity and accuracy, we will revise the curriculum to include a more straightforward question format, such as “Is this melanoma: yes or no?” This adjustment could facilitate greater understanding and potentially lead to more substantial increases in triage rates.

## Future directions

In Oregon, there are ~8,905 PCPs (32) and only 235 dermatologists (33) (1:38 ratio), highlighting the potential impact their collaboration could have on screening efforts. Given Oregon's mix of urban and rural settings, this toolkit provides PCPs with a wide selection of referral options for dermatology, including both in-person clinicians and virtual e-consults (29). The worsening access issues caused by dermatologist shortages (1) underscore the importance of enhancing PCP's screening abilities through education. To encourage these efforts, we must consider the barriers hindering PCPs from conducting skin exams, including time constraints, limited dermatologic training, and lack of confidence (8, 9). Following participation in our curriculum, PCPs identified an increased number of barriers to providing skin exams, revealing an area for improvement to address these challenges and offer practical solutions on how to perform time-efficient skin exams. Coordinated approaches, including preemptive identification of high-risk patients and integrating skin checks into physical examinations, can help alleviate time constraints (8, 30). Additionally, given the growing interest among PCPs in dermoscopy as handheld tools become more accessible, we may consider incorporating basic methods for learning dermoscopy in future iterations of this toolkit (i.e. three-point checklist, TADA method, Menzies method) (31). Furthermore, although most PCPs lack extensive dermatologic expertise, our data suggests that additional training can improve their confidence and knowledge in identifying and diagnosing skin cancer. While our study did not yet measure the amount of PCPs intending to conduct regular skin examinations post-intervention, future studies can help elucidate this change and provide insights on the behavioral impact of educational interventions. Additionally, our study focused on immediate outcomes, such as improvements in confidence and lesion identification. To better assess the long-term impact of the MTED program, future longitudinal studies should evaluate its effects on clinical behavior changes and patient outcomes, helping to validate the broader efficacy of the curriculum.

## Data Availability

The original contributions presented in the study are included in the article/Supplementary material, further inquiries can be directed to the corresponding author.
