# Correction: Melanoma toolkit for early detection for primary care clinicians: a 1-year follow-up on outcomes

**DOI:** 10.3389/fmed.2026.1827799

**Published:** 2026-03-23

**Authors:** Kyra Diehl, Elizabeth Stoos, Alyssa Becker, Victoria E. Orfaly, Jacob Nelson, Jordan Gillespie, Justin Ng, Tayler Tobey, Emile Latour, Joanna Ludzik, Elizabeth G. Berry, Alan C. Geller, Heidi Jacobe, Justin Leitenberger, Danielle McClanahan, Jessica Tran, Smriti Prasad, Stephanie Mengden-Koon, Kelly C. Nelson, Ryan Petering, Alex Verdieck, Stephanie Savory, Emily H. Smith, Susan Tofte, Martin A. Weinstock, Kevin White, Oliver Wisco, Clara Curiel-Lewandrowski, Susan M. Swetter, Alexander M. Witkowski, Laura Ferris, Samantha Black, Rebecca Xu, Shuai Xu, Sancy Leachman

**Affiliations:** 1 Department of Dermatology, Oregon Health & Science University, Portland, OR, United States; 2 School of Medicine, Oregon Health & Science University, Portland, OR, United States; 3 Biostatistics Shared Resource, Knight Cancer Institute, Oregon Health & Science University, Portland, OR, United States; 4 Department of Bioinformatics and Telemedicine, Jagiellonian University Medical College, Kraków, Poland; 5 Department of Social and Behavioral Sciences, Harvard T.H. Chan School of Public Health, Boston, MA, United States; 6 Department of Dermatology, University of Texas Southwestern Medical Center, Dallas, TX, United States; 7 Department of Dermatology, The University of Texas MD Anderson Cancer Center, Houston, TX, United States; 8 Department of Family Medicine, Oregon Health & Science University, Portland, OR, United States; 9 Department of Dermatology, University of Missouri, Columbia, MO, United States; 10 Department of Dermatology, The Warren Alpert Medical School of Brown University, Providence, RI, United States; 11 Providence Veteran Affairs Health Care System, Providence, RI, United States; 12 Dermatology Health Specialists, Bend, OR, United States; 13 Department of Dermatology, University of Arizona College of Medicine, Tucson, AZ, United States; 14 Department of Dermatology, Stanford University Medical Center and Cancer Institute, Palo Alto, CA, United States; 15 Department of Dermatology, University of Pittsburgh, Pittsburgh, PA, United States; 16 Brigham and Women's Hospital, Harvard Medical School, Boston, MA, United States; 17 School of Medicine, Northwestern University, Chicago, IL, United States; 18 Knight Cancer Institute, Oregon Health & Science University, Portland, OR, United States

**Keywords:** education, melanoma, primary care, skin cancer, skin neoplasms, training

The affiliation “Department of Bioinformatics and Telemedicine, Jagiellonian University Medical College, Kraków, Poland” was omitted for author Joanna Ludzik. Joanna Ludzik should have “Department of Dermatology, Oregon Health & Science University, Portland, OR, United States” and “Department of Bioinformatics and Telemedicine, Jagiellonian University Medical College, Kraków, Poland”.

The original version of this article has been updated.

