# Supplementary material for: Melanoma toolkit for early detection for primary care clinicians: a 1-year follow-up on outcomes
Source: Front Med (Lausanne). 2024 Dec 18;11:1500216. doi: 10.3389/fmed.2024.1500216 (PMC11688185; doi:10.3389/fmed.2024.1500216)

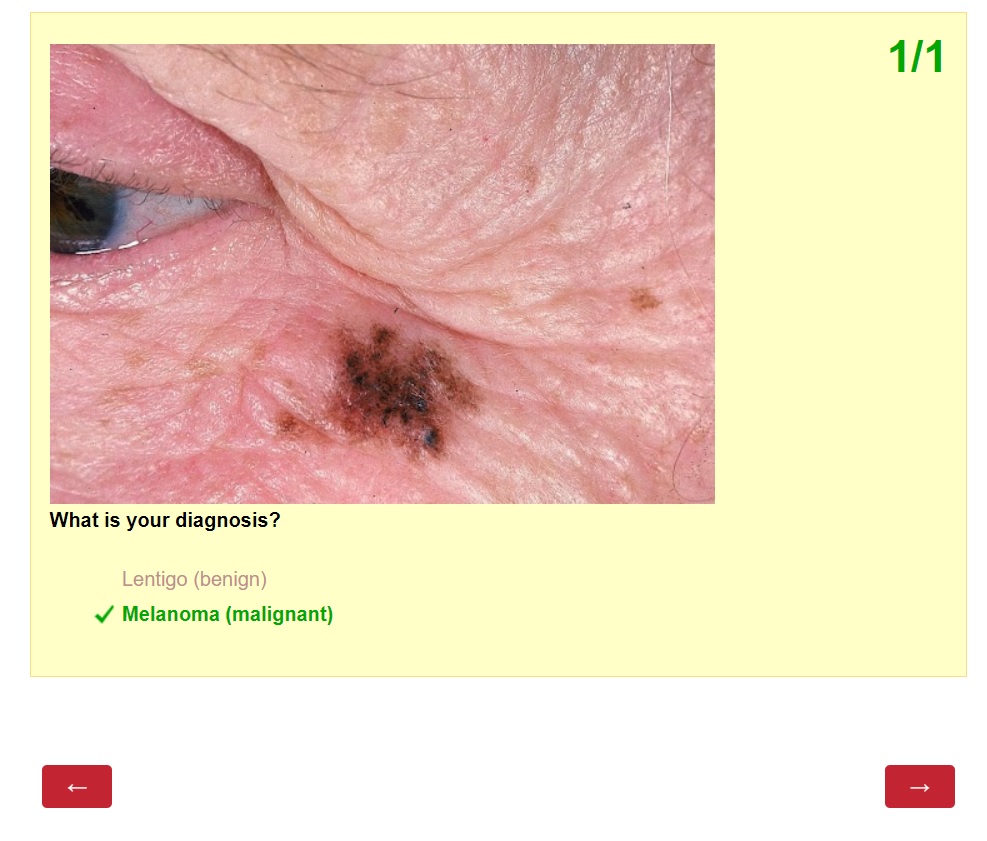


Figure 1 Practice with Immediate Feedback


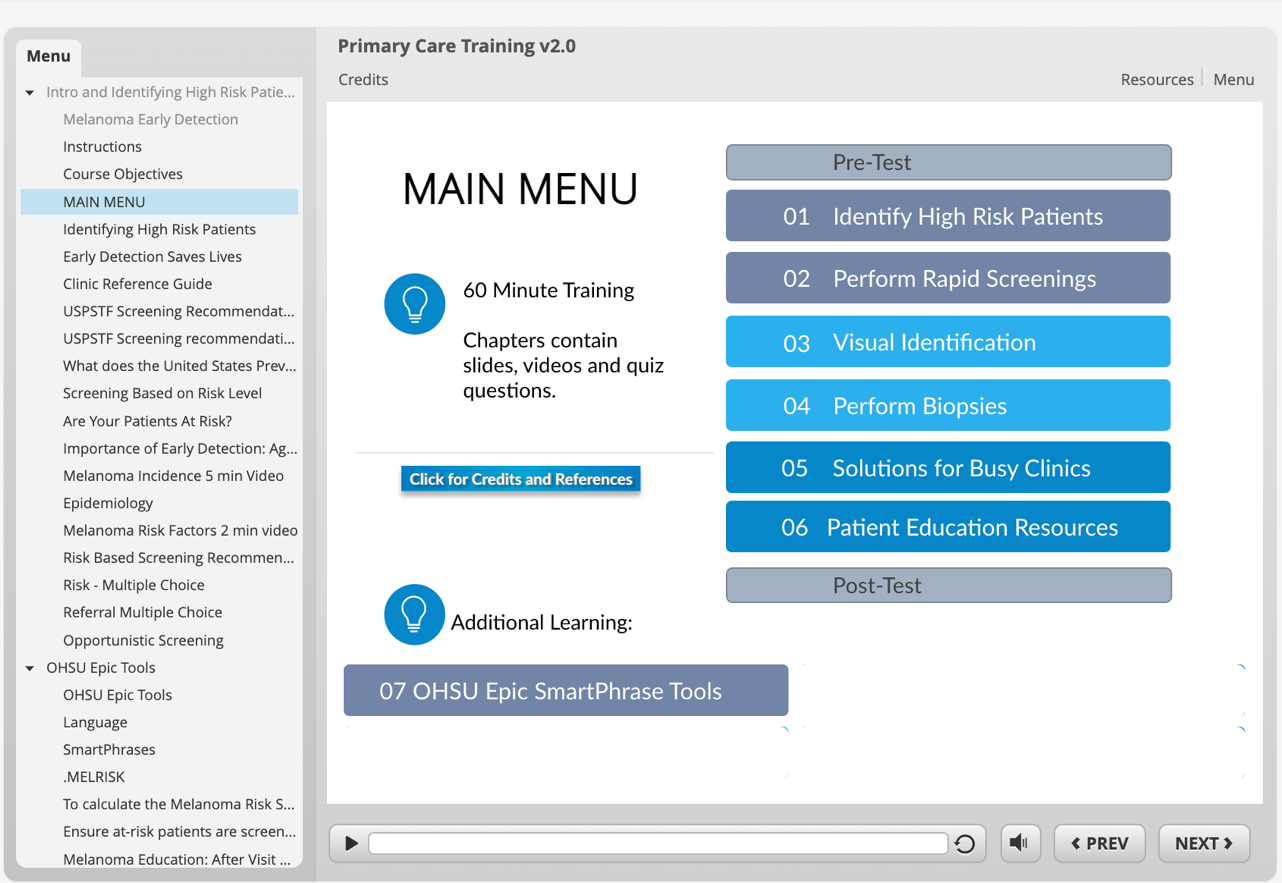


Figure 2 Self-Paced Segmented Learning


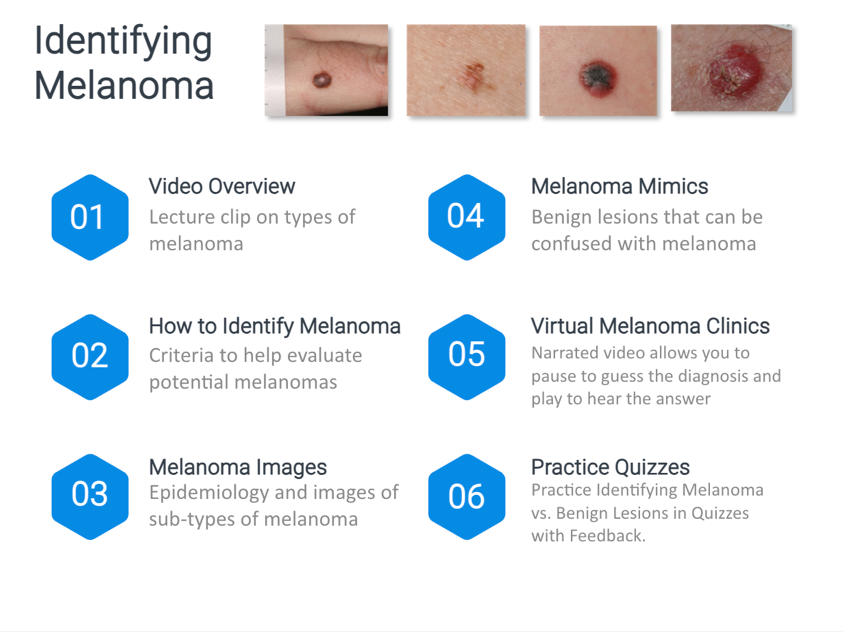


Figure 3 Image Based Active Learning


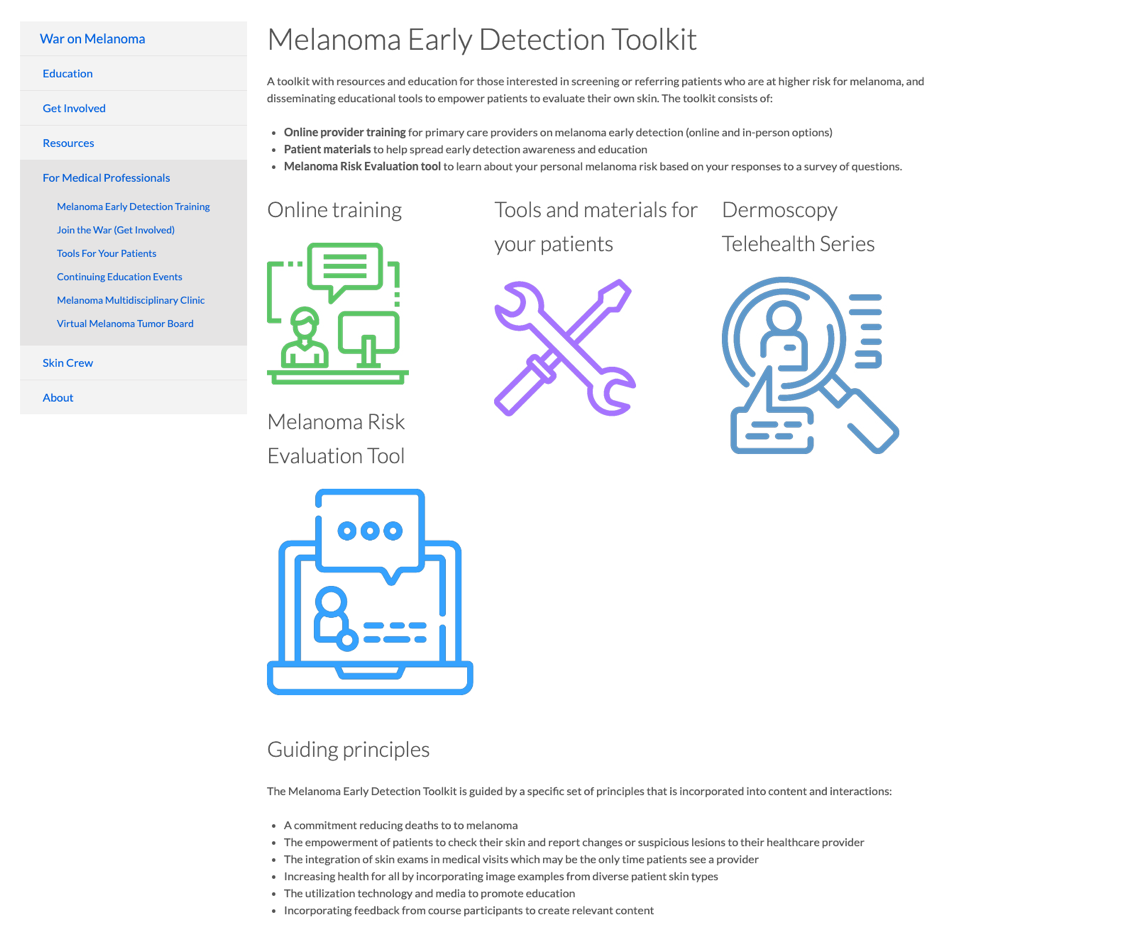


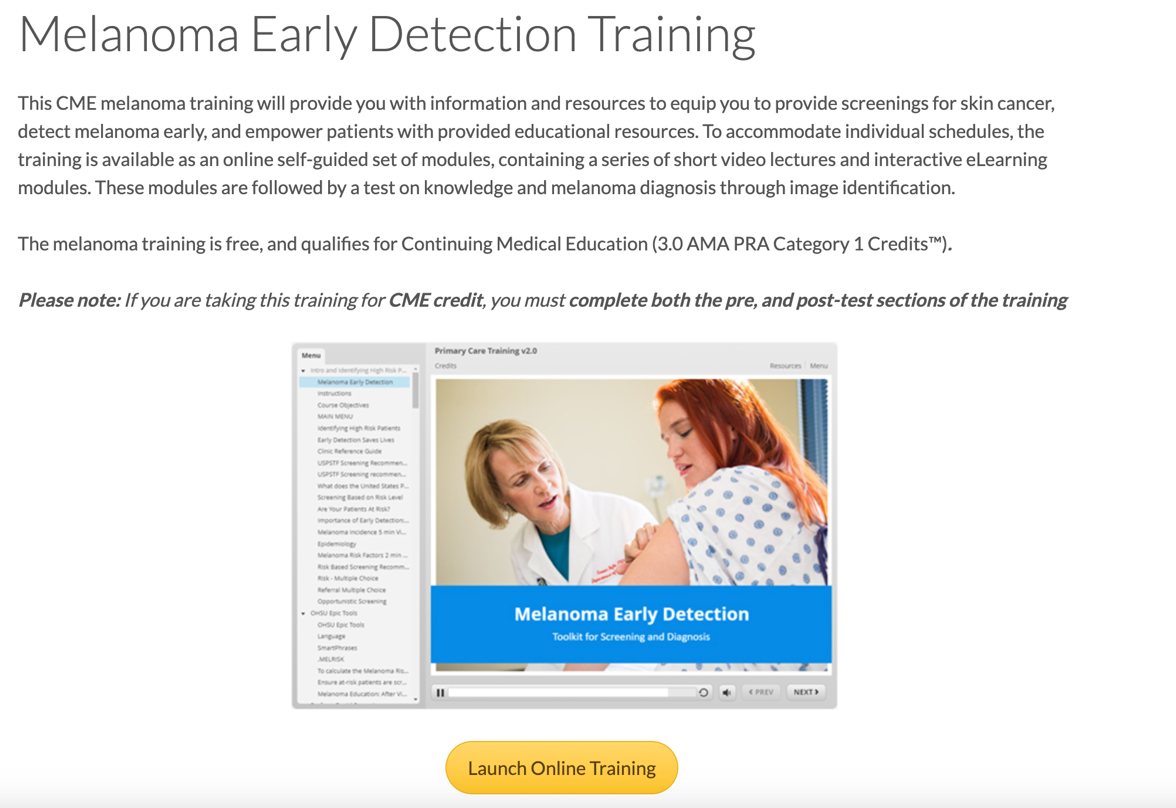


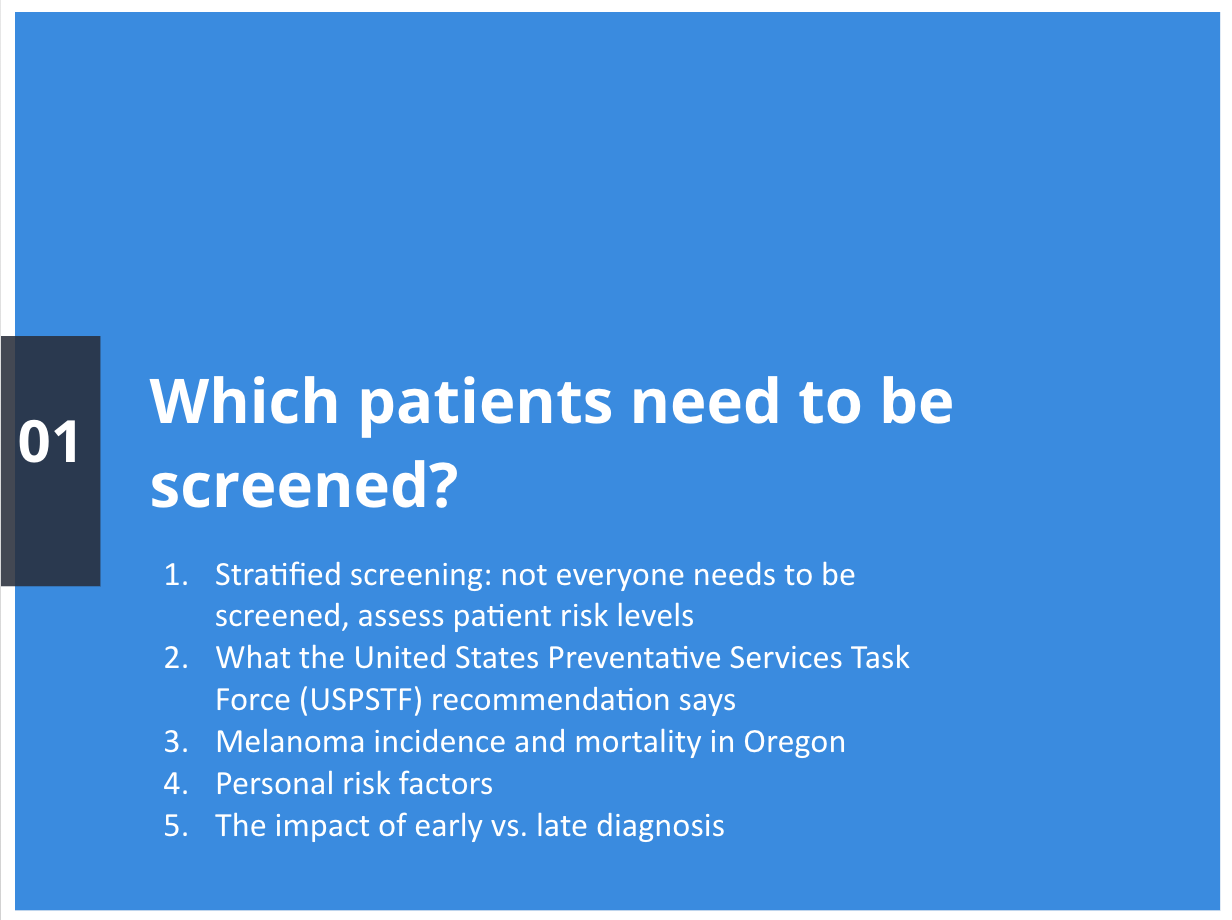


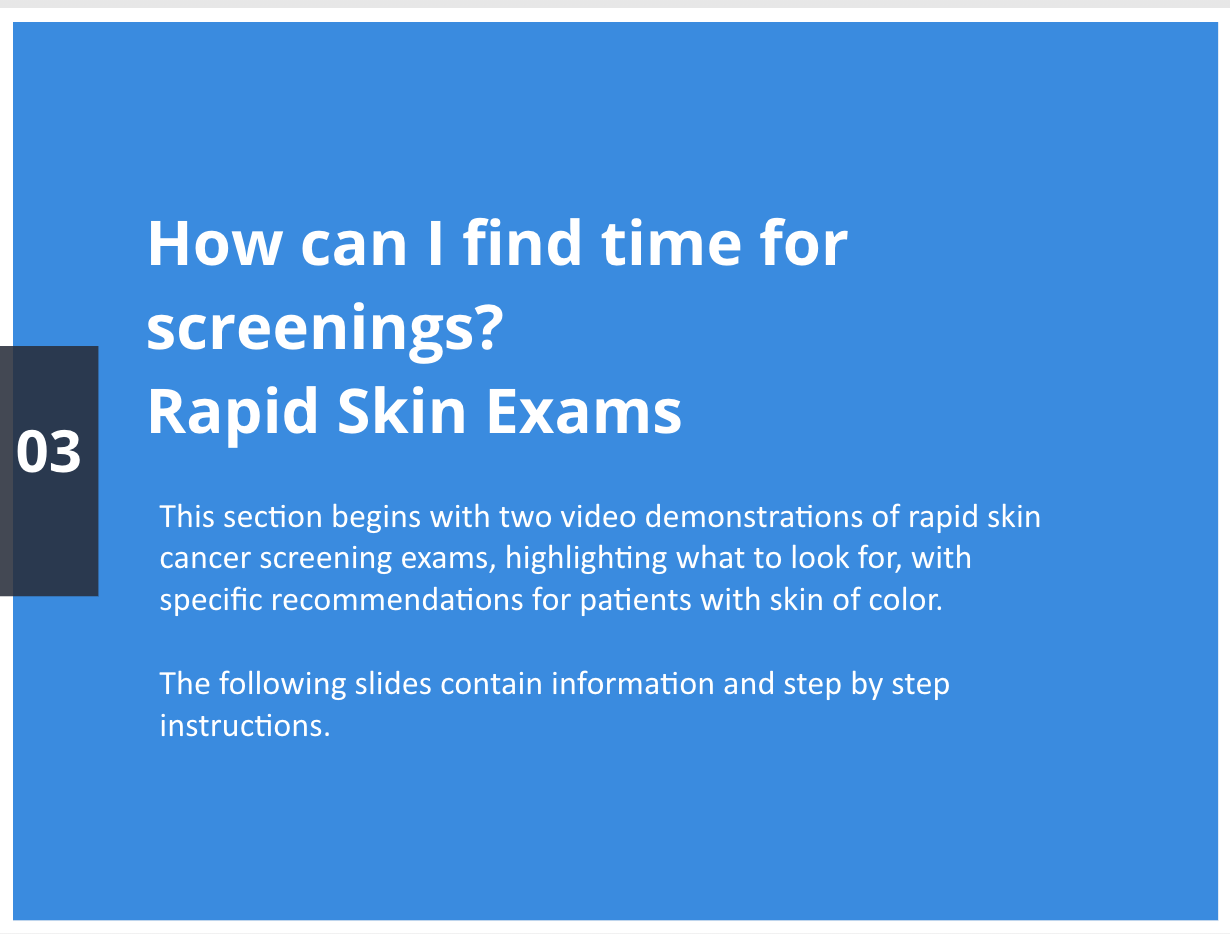


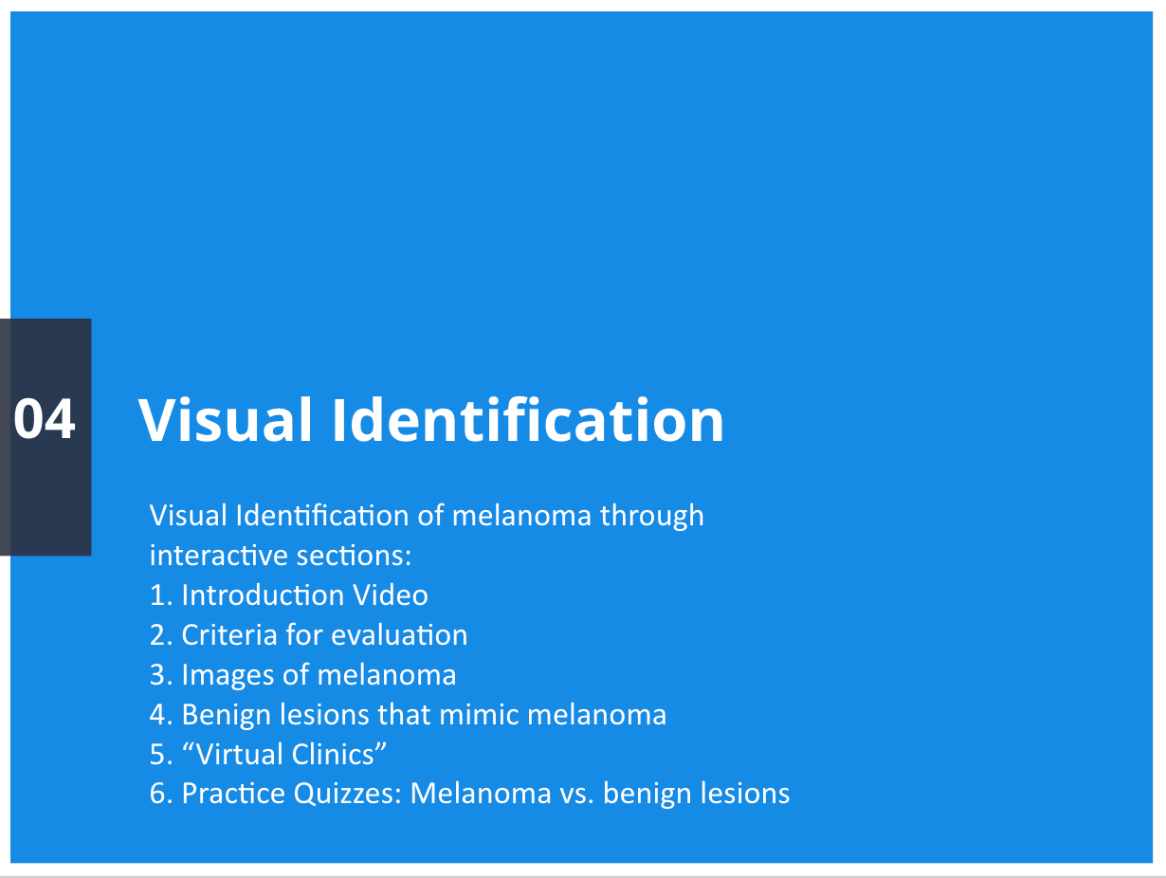


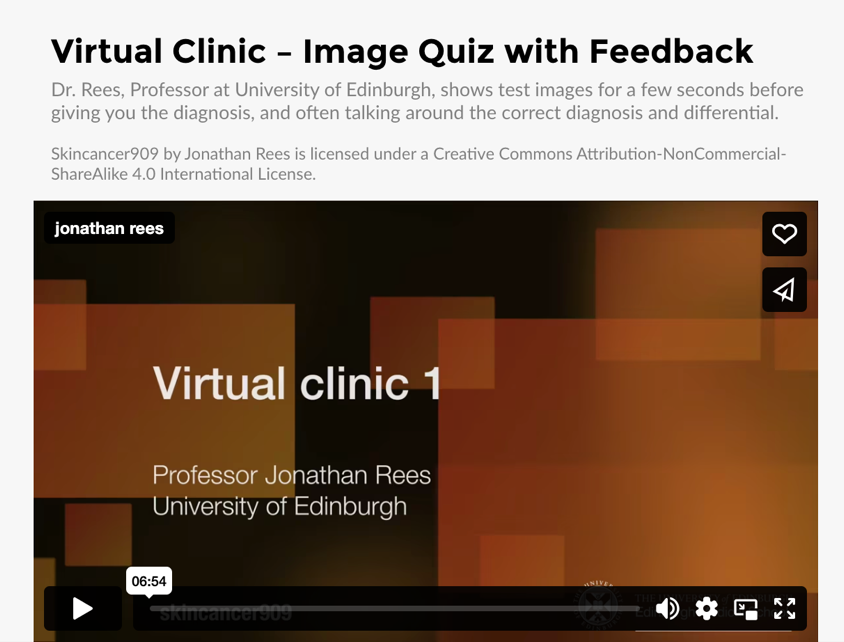


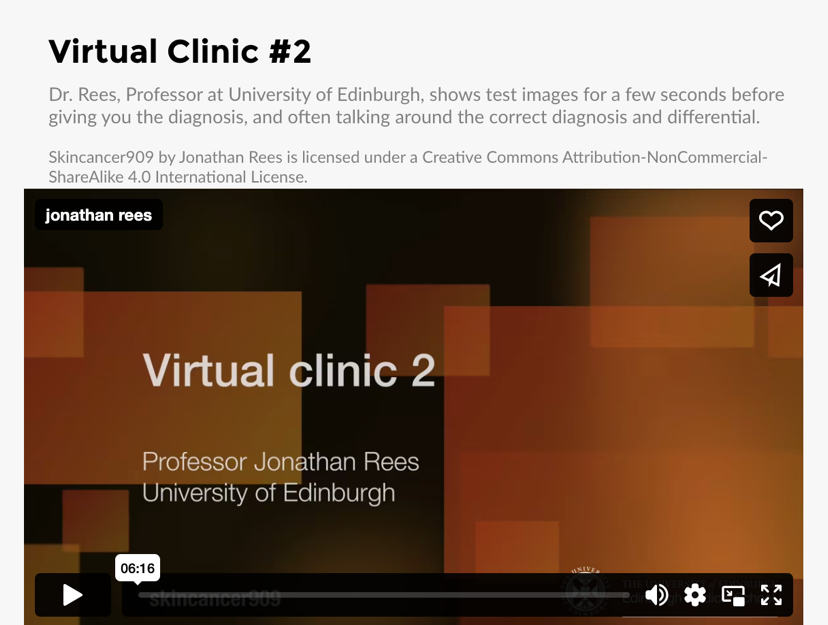


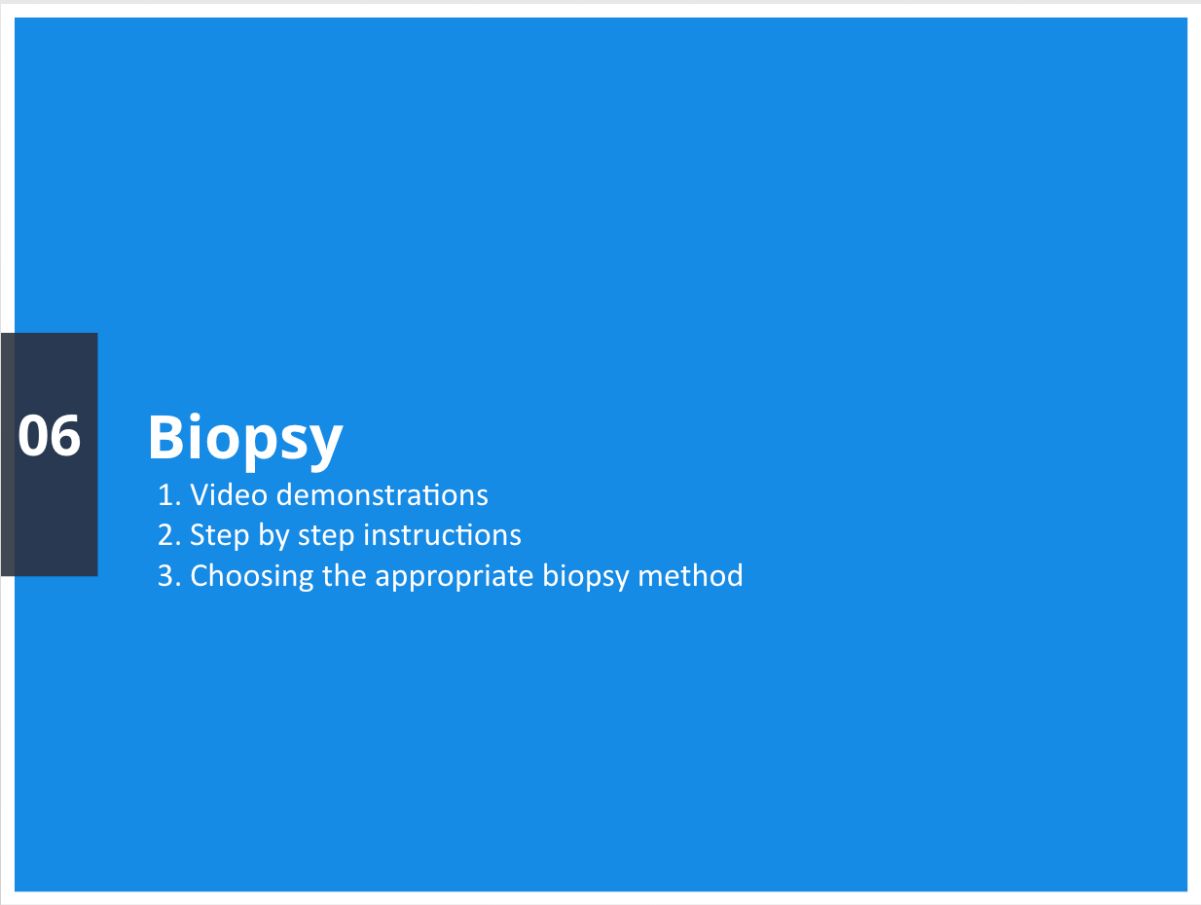


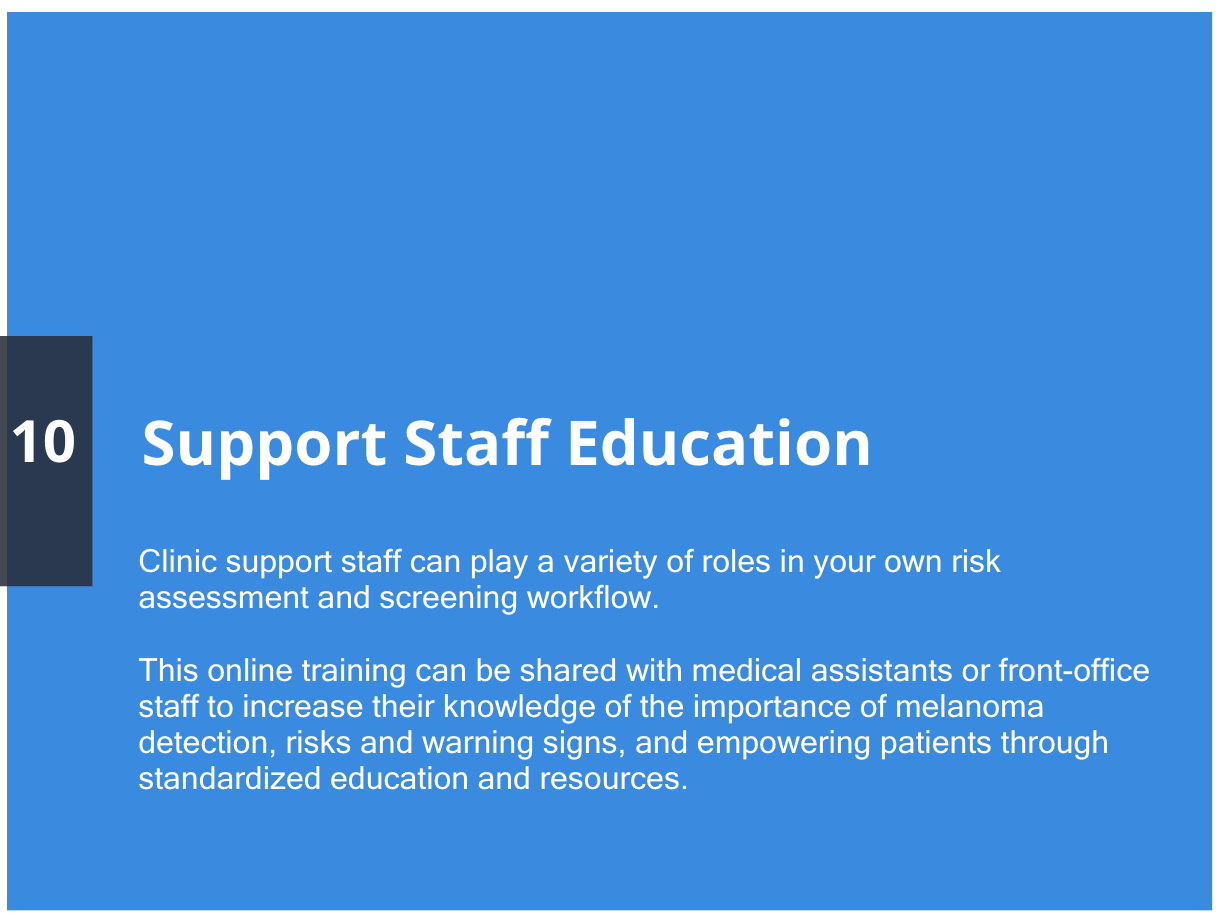

Supplement: Supplementary file 1 [file Table_1.DOCX]
